# A micropatterned substrate for on-surface enzymatic labelling of linearized long DNA molecules

**DOI:** 10.1038/s41598-019-51507-z

**Published:** 2019-10-21

**Authors:** Dharma Varapula, Eric LaBouff, Kaitlin Raseley, Lahari Uppuluri, Garth D. Ehrlich, Moses Noh, Ming Xiao

**Affiliations:** 1 School of Biomedical Engineering, Drexel University, Philadelphia, PA 19104 USA; 2 Department of Microbiology and Immunology, Drexel University College of Medicine, Philadelphia, PA 19102 USA; 3 Center for Genomic Sciences and Center for Advanced Microbial Processing, Institute of Molecular Medicine and Infectious Disease, Drexel University College of Medicine, Philadelphia, PA 19102 USA; 4 Department of Otolaryngology Head and Neck Surgery, Drexel University College of Medicine, Philadelphia, PA 19102 USA; 5 Department of Mechanical Engineering and Mechanics, Drexel University, Philadelphia, PA 19104 USA

**Keywords:** Biophysical methods, Lab-on-a-chip, Lab-on-a-chip, Lab-on-a-chip, Biophysical methods

## Abstract

Optical mapping of linearized DNA molecules is a promising new technology for sequence assembly and scaffolding, large structural variant detection, and diagnostics. This is currently achieved either using nanochannel confinement or by stretching single DNA molecules on a solid surface. While the first method necessitates DNA labelling before linearization, the latter allows for modification post-linearization, thereby affording increased process flexibility. Each method is constrained by various physical and chemical limitations. One of the most common techniques for linearization of DNA uses a hydrophobic surface and a receding meniscus, termed molecular combing. Here, we report the development of a microfabricated surface that can not only comb the DNA molecules efficiently but also provides for sequence-specific enzymatic fluorescent DNA labelling. By modifying a glass surface with two contrasting functionalities, such that DNA binds selectively to one of the two regions, we can control DNA extension, which is known to be critical for sequence-recognition by an enzyme. Moreover, the surface modification provides enzymatic access to the DNA backbone, as well as minimizing non-specific fluorescent dye adsorption. These enhancements make the designed surface suitable for large-scale and high-resolution single DNA molecule studies.

## Introduction

Optical mapping of genomic DNA is increasingly becoming an essential tool in whole genome analyses and in detection of structural variations (SVs). To achieve unambiguous and complete genome sequence assembly using high-throughput short-read sequencing, long-range sequence information is necessary. This is currently obtained by use of either or both, optical mapping and long-read sequencing (PacBio and Oxford Nanopore Technology). Optical mapping is one of the preferred techniques to perform genome assembly through highly repetitive regions^1–3^, for SV detection in large human populations^4^, as well as in applications of bacterial strain-typing^5,6^ and complex SV detection and analysis^7^ among others^8,9^.

Single molecule linearization forms the basis for most optical mapping techniques. They may be characterized by the length of probed DNA molecules, throughput, and the choice of sequence-specific chemistry. Longer linearized DNA molecules deliver larger scaffold sizes for genome assembly resulting in a more rapid and accurate genome assembly. Higher throughput in optical mapping directly affects the overall time required to assemble the genome. Fluorescent labelling offers a straightforward method to visualize specific sequences, and in some cases offers multiple layers of mapping information^9,10^. Currently, there are two commercial optical mapping technologies, Bionano Genomics’ Irys and Saphyr, and OpGen’s Argus. While the former has higher throughput and uses fluorescent labelling chemistry, the latter is successful at probing longer DNA molecules. Besides the above two techniques, several models have been developed that can linearize a DNA molecule to visualize specific sequences.

DNA linearization techniques can be broadly classified into two types, DNA adsorption on a solid surface^11,12^ and nanoscale confinement^13,14^. Physical adsorption of DNA has been realized either through hydrophobic-hydrophobic interaction^15^, termed molecular combing, or via electrostatic forces^12^. Molecular combing is a powerful technique owing to relatively uniform extension, high DNA density^16^, and high average DNA length^17^. Combed DNA molecules on a hydrophobic substrate are typically extended to 115–145% of their contour length, depending on the hydrophobicity of the surface^11^. Overstretched DNA has been shown to not interact efficiently with sequence-recognizing enzymes^18^, thereby limiting sequence-specific detection to hybridization chemistry^16^. On octenyl-coated substrates (common hydrophobic modification), long human genomic DNA (hgDNA) molecules (up to multiple Mbp) often agglomerate, cross paths during linearization, or form loops due to adsorption of both DNA ends in close proximity (producing DNA loops)^19^. Moreover, hydrophobic substrates have high binding affinities for the vast majority of fluorophores^20^. In some variations of molecular combing, DNA molecules have been sequence-specifically labelled prior to linearization on a surface^21–23^. However, fluorescent background due to nonspecific dye nucleotides has not been addressed and could potentially prevent adoption for large-scale genomic DNA studies. DNA stretching via electrostatic interaction has been adapted to single molecule optical mapping by Schwartz *et al*. (1993). Although DNA was not overstretched to allow sequence-recognition by restriction endonucleases, DNA extension uniformity and throughput are not comparable to that achieved with molecular combing. In one instance, fluorescently labelled T7 DNA was linearized on a positively charged amine surface. However, this required purification of the labelled DNA sample and the compatibility of the amine surface for low-fluorescence background was not reported^24^. In comparison, polyelectrolyte-coated glass substrates have been used to efficiently linearize fluorescently-labelled DNA molecules without purification^25,26^. Thus, no study has been reported to date that has demonstrated on-surface enzymatic labelling of adsorbed DNA.

DNA elongation in nanochannels provides more uniform linearization compared to molecular combing. However, nanoscale fabrication is a significant cost factor in deploying this technology, and due to limited nanochannel real estate, several cycles of DNA loading and imaging are necessary to obtain sufficient coverage for genome assembly. Fluorescent labelling of DNA molecules confined in a nanochannel is not feasible due to the suppressed diffusion kinetics. Typically, DNA is labelled in a test tube before being loaded into nanochannels for imaging. This systematic limitation could potentially restrict the amount of information that can be obtained from single molecules^27^. In contrast, linearizing DNA molecules on an open, solid surface is a one-step, straightforward process, making the technique limited only by image acquisition speeds. More importantly, multiple enzymatic reactions may be performed sequentially to obtain detailed information.

The DNA curtain assay technique^28^ uses a diffusion barrier to anchor DNA that is fluid within a lipid bilayer, and then stretch the DNA molecules under controlled flow. This technique allows for real-time fluorescence studies of DNA-protein binding unlike any of the above. This technique, however, still relies on modifying the DNA ends before linearization. Moreover, it has not been shown to be able to interrogate Mbp-long DNA molecules.

We have developed a micropatterned substrate with two contrasting surface functionalities, one region grafted with octenyl and the other with polyethylene glycol (PEG) moieties. The ends of a coiled DNA molecule selectively attach to the octenyl section and majority of its backbone spans the passivating PEG section. The PEG sections support enzymatic access and minimize adsorption of fluorescent dye-nucleotides. By modulating the contact angle of receding meniscus via PEGylation, non-overstretched DNA molecules were obtained. We first performed on-surface transcription on T7 DNA molecules to confirm sequence-recognition by enzyme. To show on-surface sequence-specific fluorescent labelling of linearized DNA, we combed λ-DNA on a micropatterned substrate and performed nick-labelling^25^ in a hydrogel environment. Randomly picked labelled-λ-DNA molecules aligned to the reference map readily, demonstrating the device’s potential for sequential labelling chemistries and real-time base-by-base incorporation.

## Results and Discussion

Our micropatterned surface is dual-functionalized, with two repetitive functional areas. One area is functionalized with octenyl, which is hydrophobic and adsorbs the tail-ends of DNA molecules. The other area is functionalized with polyethylene glycol (PEG), a passivating group which does not attract DNA and suppresses the attachment of free stain and dye-nucleotide molecules. With this micropatterned surface, DNA molecules bind in an end-selective manner to the hydrophobic octenyl surface only, and then linearize through PEG regions by receding meniscus through dynamic combing. DNA molecules can be stretched in an orderly fashion with less potential for formation of both intermolecular intersections and intramolecular loops.

### DNA adsorption on octenyl and amine surfaces

For DNA combing to work on this micropatterned surface, DNA ends need to preferentially bind to the octenyl sections. Dynamic molecular combing (coverslip withdrawn from a reservoir) is the most widely used method of generating such receding menisci among others including gravity^29,30^, dragging^31^, capillary flow^26^, wicking with filter paper^32^, and evaporation. We first compared DNA adsorption and linearization on octenyl and amine-grafted surfaces. Parallel, linear individual molecules adsorbed to octenyl surface in an orientation perpendicular to the receding meniscus, while on amine surface, DNA molecules were found to be adsorbed in a globular form (data not shown). This is consistent with the fact that a coiled DNA molecule was expected to adsorb at multiple points along its backbone through the electrostatic attraction between the negatively charged DNA backbone and the weakly cationic amine moieties. In order to linearize the DNA molecules on an amine surface, a concurrent shearing flow was necessary to generate linearization at an adequate rate compatible with the adsorption kinetics between DNA and alkylamines^33^. Clearly, preferential attachment of DNA ends to octenyl surface is critical for dynamic combing.

### Fabrication parameters affecting DNA attachment

**Figure 1:**
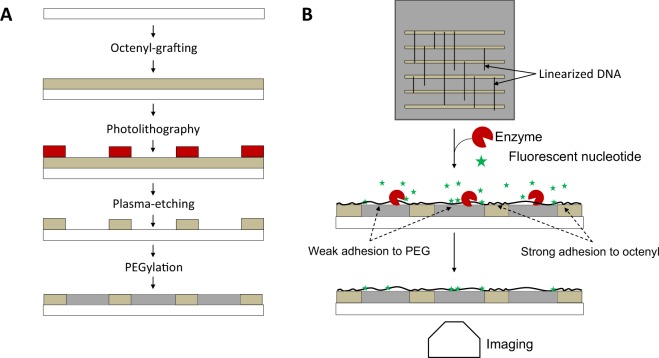
Micropatterning & dual-functionalizing glass substrates for DNA linearization. (**A**) Fabrication process flow employed in micropatterning glass substrates; octenyl sections are 18 mm long and 10 to 40 μm wide; PEG sections are 10 to 170 μm wide. (**B**) Individual DNA molecules that selectively end-adsorb to octenyl sections are subsequently linearized through traditional molecular combing with a receding meniscus. Molecules linearize across passivated, PEG sections. Upon linearization, the DNA molecules may be modified using enzymatic labeling chemistries including fluorescent dye incorporation.

Our micropatterned octenyl/PEG surface was designed in part to alleviate the complications of DNA combing, such as DNA aggregation and high fluorescent background of the hydrophobic silanized substrate. Figure 1A shows a schematic of such a micropatterned surface. A “binding region” (olive-green) on the substrate is silanized with the octenyl functional group to promote DNA end-attachment. The “extending region” (grey) is functionalized with PEG for DNA linearization, on-surface modification, and observation. The spatial ratio between these two regions can be controlled to select for a targeted molecular size and to control combing density for fewer intermolecular crossing events and reduced intramolecular loop formation for superior observation and interrogation conditions.

To characterize the silanization process, we measured contact angles on octenyl and PEG regions using Surface Analyst 3001 (BTG Labs). When glass substrates were grafted with octenyl (reaction time, 4 h) following surface activation using air-plasma (200 W, 3 min), the average contact angle (triplicate) was 73°. Piranha-activation followed by overnight silanization resulted in substrates with marginally higher hydrophobicity (average contact angle, 76°) but this was accompanied with reduced reproducibility. Contact angle measured on a *10–10* micropatterned substrate after PEG-grafting (2-[methoxy(polyethyleneoxy)6–9propyl]trimethoxysilane (PTMS), 32.5 nM) was found to be 26° in the PEG-only region and 45° in the patterned region. Different contact angles confirm the presence of contrasting surface functional groups, octenyl and PEG. Interestingly, contact angle on the *10–10* substrate, that has an even distribution of the two modifications, was in between the contact angles on octenyl and PEG-coated surfaces.

Photolithography soft-bake (SB) temperature as well as PTMS concentration were found to affect DNA attachment density. Due to inconsistent surface quality after silanization, a higher SB temperature was initially necessary to prevent occurrence of ‘mouse bites’ (poor PR adhesion). To assess the impact, photolithography was performed on two octenyl-coated glass substrates with SB temperatures, 95 and 115 °C. PR was stripped, and substrates were cleaned thoroughly before combing T7 DNA followed by visualization. For the substrate baked at 115 °C, DNA density was observed to be lower in the previously UV-unexposed region (refer Fig. 1A) than in the exposed region. However, the 95 °C-baked substrate had similar DNA densities on both, previously UV-unexposed and UV-exposed regions (data not shown). After achieving consistent octenyl surface coatings, an SB temperature of 95 °C resulted in crisp micropatterns. This interaction between unexposed PR (SC1813) and octenyl functional group (or any silane) at 115 °C has not been reported previously.

To ascertain that the PR thin film effectively shielded underlying octenyl layer from plasma treatment, T7 DNA was combed onto micropatterned substrates that were plasma-treated, PR-stripped and cleaned. DNA combing density on the octenyl region remained unaffected compared to that observed on substrates that were not treated with plasma. Moreover, there was no DNA attached to the activated glass surface, indicating a high degree of hydrophilicity.

The PTMS concentration we found to be optimum was 32.5 nM. At higher concentrations (>240 nM), DNA combing density was found to decrease dramatically, likely due to a parallel reaction with unreacted methoxy groups (or hydroxyls) in the octenyl region. Higher DNA concentration (3x) in the combing reservoir did not improve DNA density significantly.

### DNA linearization on micropatterned substrate

**Figure 2:**
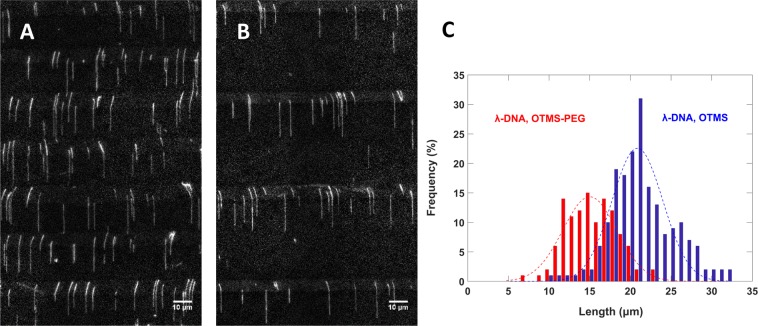
Combing λ-DNA on micropatterned, dual-functionalized glass. λ-DNA molecules were combed onto *10–15* (**A**) and *10–40* (**B**) substrates. Octenyl sections appear as bright strips due to adsorption of YOYO-1 dye in contrast to the low-fluorescence background of PEG sections. Molecules were predominantly end-bound to the octenyl sections and extended across the PEG sections. Both, *10–15* and *10–40* resulted in similar linearization, with relatively lower DNA density on the *10–40*. (**C**) Overlaid histograms with matching Gaussian regressions reveal the length distributions of λ-DNA molecules combed on unpatterned octenyl substrate (blue, n = 191) and micropatterned octenyl-PEG (red, n = 116). octenyl-PEG substrate produced significantly lower DNA extension.

A micropatterned glass substrate with 10 μm-wide octenyl and 15 μm-wide PEG sections (*10–15*) was combed with λ-DNA. The substrate was immersed into the DNA solution for an extended incubation time (compared to an unpatterned octenyl substrate) of 15 minutes, after which it was withdrawn at 0.1 mm/s, dip-stained in a reservoir containing YOYO-1, and imaged (Fig. 2A). The octenyl sections appeared brighter than adjacent PEG sections, due to adsorption of YOYO-1. On an average, over 98% of combed DNA molecules extended with one end bound to the upper octenyl section. It is interesting that we observed very few molecules with two ends attached to the same octenyl section forming a loop. Similar results were obtained with λ-DNA linearized on a *10–40* substrate although with lower combing density due to reduced effective area of binding (Fig. 2B). DNA molecules with a single end bound to octenyl still linearized into the PEG section (Fig. 2B), suggesting adhesion to PEG region, albeit substantially weaker than on octenyl sections. High power 473 nm laser illumination on YOYO-1-intercalated combed DNA resulted in visible DNA breaks on octenyl-PEG substrates leaving behind DNA fragments tethered to the few attachment points across the PEG sections (data now shown). This observation was similar to the one reported by Gueroui *et al*. on rehydrated polymethylmethacrylate (PMMA) coated glass^18^. Linearized molecules in the octenyl sections appeared bent, but no such bends were observed on molecules extended in the PEG section. It has been shown earlier that the orientation of combed DNA is directly affected by the shape of the receding meniscus^34^. The meniscus in the polypropylene DNA reservoir was concave in shape and likely contributed to the bends. In addition, it has been shown that a micropatterned surface (with hydrophobic-hydrophilic alternating regions) could affect the shape of the receding meniscus, and hence the orientation of DNA deposition^35^. Also, the appearance and degree of DNA bends was significantly more away from the centre of the substrate than at the centre. Hence, we surmise that the bends were due to the unique motion of receding meniscus, a combination of both the factors.

To evaluate the stretching factor (s.f., ratio of measured and true contour length) of DNA on octenyl-PEG substrate, λ-DNA was combed on a *10–40* substrate as well as on an unpatterned octenyl substrate. DNA backbone length measurements on the unpatterned substrate yielded a peak at 21 μm (Fig. 2C, blue), corresponding to an s.f. of 127%. For the *10–40* substrate, backbone lengths were measured separately for each silanized section, noted as l_octenyl_ and l_PEG_. The histogram plotted for the combined length, l_overall_ (l_octenyl_ + l_PEG_), and fitted with a gaussian curve, yielded a peak at 14.7 μm (Fig. 2C, red), which corresponds to an s.f. of 89%. Further, on the *10–40* substrate, we assumed 127% stretching in the octenyl section, and derived s.f. on the PEG section using the equation below.

$$s.f{.}_{PEG}=\frac{{l}_{PEG}}{(16.49-(\frac{{l}_{octenyl}}{1.27}))}$$

Resulting mean s.f. for PEG sections were found to be ~84%. This clearly reflects the overall reduction in s.f. due to PEG surface modification. By increasing density of the grafted PEG, we may potentially be able to under-stretch the DNA further. In general, the micropatterned substrates produced marginally higher stretching uniformity compared to unpatterned octenyl substrates, with standard deviations (S.D.) of 3 µm and 4.1 µm, respectively. Additionally, individual molecules were observed to be less aggregated on octenyl-PEG substrates compared to unpatterned octenyl substrates. Labit *et al*., based on which our octenyl-silanization process was designed, reported a peak length between 21 and 23 μm for λ-DNA combed on unpatterned octenyl substrate. Vapor-phase-silanized octenyl substrates too have been shown to have identical mean length (21.5 μm), but lower S.D. (0.5 μm)^15^. Other hydrophobic polymer-coated surfaces such as polystyrene^11^ and Zeonex^36^ have been shown to stretch λ-DNA to a mean length of 27 and 27.6 μm (S.D. 1.2 μm) respectively.

**Figure 3:**
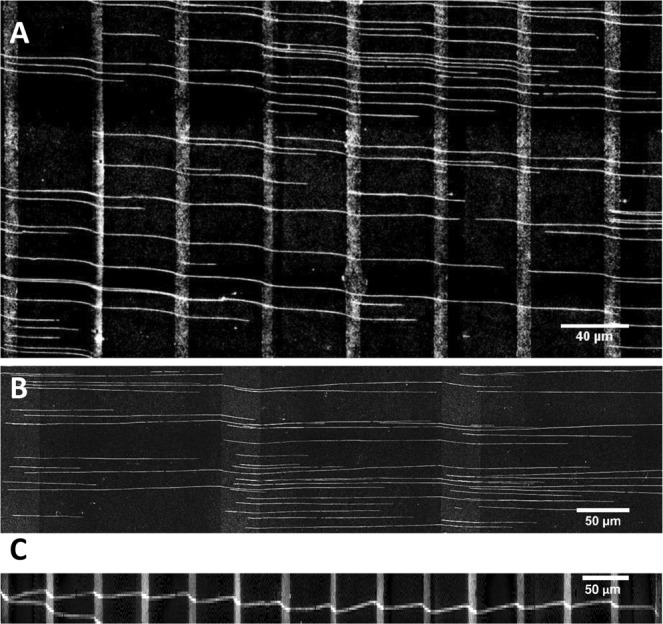
Combing hgDNA on various patterns. High molecular weight human DNA was combed on micropatterned octenyl-PEG substrates to demonstrate the surface’s ability to adsorb and isolate long molecules and to explore the significance of pattern design parameters in combing long molecules. Human DNA combed on two patterns are shown here: (**A**) *10–40* and (**B**) *40–170*. As in Fig. 3, molecules were seen to absorb to the octenyl sections in an end-selective fashion; (**C**) An example image of human DNA >2 Mbp long combed onto a *10–40*.

We then proceeded to investigate linearization of long human genomic DNA (hgDNA) molecules onto octenyl-PEG substrates. Typical resulting images are shown in Fig. 3. As shown in Fig. 3A, the tail ends of long hgDNA preferentially bound to octenyl sections of the *10–40* substrate. Of 326 molecules measured, fewer than 24 had the leading end bound to a PEG section rather than an octenyl section. The combed hgDNA molecules were also more orderly, with very few molecules crossing each other. Fewer loops were observed compared to combing on an unpatterned octenyl substrate, possibly due to the reduced chance of dual-end binding events occurring in a given 10 μm octenyl section. Figure 3B shows similar combing results on a *40–170* substrate, with lower binding density. Table 1 summarizes the average lengths of combed DNA on *10–40* and *40–170* substrates, calculated with a minimum length threshold set at 100 kbp. Here, we used the s.f. value obtained from λ-DNA measurements on *10–40* substrate to calculate the average lengths in kbp. On the *10–40* substrate, 84.42% of the molecules were longer than 300 kbp with average at 677 kbp, and over 20% of them were above 1 Mbp in length. DNA molecules combed on the *40–170* substrate were generally longer, with 32.4% over 1 Mbp. We routinely observed very long (>1 Mbp) molecules using these longer pitch micropatterned substrates. One DNA molecule approximately 2 Mbp long is shown in Fig. 3C. It is not clear why a greater percentage of long molecules were observed on the *40–170* substrate. We speculate that this is likely a result of the combination of reduced binding area and difference in adsorption strengths of long and short DNA fragments. End binding-unbinding rates of a small DNA molecule is likely higher (lower adhesion strength) than that of a longer molecule, due to the lower number of available binding points required to stabilize adsorption^37^. Because end-binding of DNA molecules occurs only during incubation and not during recession of the meniscus, the bias towards adsorption of longer DNA fragments likely increases. A more careful investigation into this will be pursued in our future work.

**Table 1:** Molecular size distribution of human DNA combed on *10–40* (10 μm binding, 40 μm passivating) and *40–170* (40 μm binding, 170 μm passivating) micropatterned glass.

Lower threshold for measured DNA length (kbp)	*10–40* substrate		*40–170* substrate	
	Percent number of molecules	Mean length (kbp)	Percent number of molecules	Mean length (kbp)
300	84.42	677.96	86.91	783.2
500	60.75	887.84	67.06	1045.38
1000	20.00	1460.91	32.40	1601.9

Molecules longer than 300 kbp were measured and counted on both substrates.

### Efficient enzymatic reactions on PEG-passivated surface

**Figure 4:**
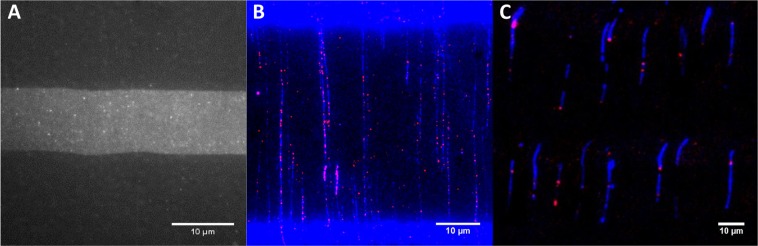
Characterization of low-fluorescence background; on-surface nick-labelling of hgDNA; on-surface transcription on T7 DNA. (**A**) A magnified image of an octenyl section and adjoining PEG sections (*10–40*) shows suppressed binding of ATTO-532-dUTP (150 nM) to PEG compared to octenyl. (**B**) ATTO-532-dUTP was successfully incorporated into combed hgDNA molecules using nick-labelling chemistry. (**C**) Transcription reaction performed using T7 RNAP on combed T7 DNA resulted in bright, labelled RNA aggregates detected along the T7 backbone.

The octenyl-PEG substrates when viewed on epifluorescence microscope at high intensity illumination (473 nm, 100–150 mW; 532 nm, 150–500 mW) barely presented any autofluorescence to enable distinction between the PEG and octenyl sections. As noted above, YOYO-1 dye molecules preferentially adsorb to octenyl sections relative to PEG sections. To further verify a reduction in adsorption of fluorescent dyes in the PEG sections, a micropatterned *10–40* substrate was incubated with a solution containing ATTO-532-dUTP (100 nM). After washing out the free dye-nucleotides from the surface, the fluorescence intensity in the octenyl section was found to be about fifteen times higher than in the PEG section. One can easily observe more distinctive bright spots in octenyl sections (Fig. 4A). This may have been due to hydrophobic-hydrophobic interactions between fluorescent moieties and the octenyl group compared to their non-interaction with the electrically-neutral and hydrophilic PEG moieties^38^.

We then tested RNA transcription of T7 DNA on our micropatterned surface. Previously, Gueroui *et al*. demonstrated on-surface transcription on a PMMA-coated glass surface, which was shown to be sequence-specific and observable only on non-overstretched DNA. In their study, an evaporating oil, 1-dodecanol, was used to obtain non-overstretched DNA molecules (~100% s.f. for T7 DNA). When replicated in our lab, we observed dodecanol residue after combing, which did not evaporate over time at room temperature or when oven-dried (65 °C) for 4 min. Moreover, reusing the same DNA reservoir with a floating dodecanol layer was not practical. By manipulating the common interface between DNA solution, combing substrate and air (triple-phase contact line) via surface modification, we achieved high density combing of non-overstretched T7 DNA. After DNA combing, we were able to perform transcription on a *10–15* octenyl-PEG substrate. RNA polymerase enzyme was mixed with fluorophore-tagged NTPs and introduced on top of combed DNA to synthesize the RNA transcripts extending from the T7 promoter sites. After the reaction time, the excess dye-NTPs were washed away and the substrate was imaged. The colocation of dye-NTP (bright red spots) and DNA backbone confirmed the presence of RNA transcripts, and hence the activity of T7 RNAP. We observed up to 4 labels per T7 DNA molecule. Notably, no transcripts (labels) were detected in the octenyl region of the substrate, likely due to overstretching of DNA. The results showed T7 RNAP successfully interacted with DNA molecules and was able to locate the promoter sites to initiate transcription (Fig. 4C). Using an in-built blending plugin, the background from octenyl sections has been suppressed. Some of the DNA molecules (blue) exhibited anywhere from 1 to 4 bright spots (red). To confirm T7 RNAP was indeed the reason for labelling, control experiments were done in parallel following the exact same procedures and using all the same reagents besides T7 RNAP enzyme. No labels were observed in any of the control experiments.

To test if two successive enzymatic reactions could be performed on the micropatterned substrate, we performed nick-labelling^25^ on hgDNA molecules linearized on a *10–40* substrate (Fig. 4B). Nick-labelling consists of two consecutive reactions – nicking with Nt.BspQI for 1 h at 37 °C followed by labelling with DNA Pol I for 1 h at 37 °C. After each reaction, the surface of the microliter-well was washed gently to remove the enzyme and free dye-nucleotide molecules. The substrate was then imaged for ATTO-532 and then for YOYO-1, and the two images superimposed to form a composite image. Most of the aggregated red spots (ATTO-532-dUTP) were observed along the blue DNA backbone in the PEG section (Fig. 4B). Much fewer free dye molecules randomly adsorbed outside the DNA backbones, indicating dye-nucleotide incorporation. Multiple long labelled DNA molecules that spanned across the 40 μm (equivalent to ~138.4 kbp) PEG section were observed in every image. In a control experiment with the same conditions but without the nickase (Nt.BspQI), we observed minimal fluorescent labelling along the DNA backbone (data not shown). This shows that both enzymes were active on the PEG surface and can be used successively. Although the reactions were found to be efficient across several trials in separate microliter-wells, the amount of combed DNA on the surface depleted significantly in most of the wells, particularly post nicking reaction (data not shown). To circumvent this, we thereafter, cast a layer of polyacrylamide (PA) gel atop the combed DNA, before proceeding with any chemical reaction. The PA layer not only helped fix the DNA by minimizing harsh physical flow forces during handling but also provided a consistently aqueous environment to conduct reactions.

Taken together from the above experiments, the PEG sections not only significantly reduce the random adsorption of free fluorescent dyes but are also amenable to successive enzymatic reactions providing for complex labelling strategies which can be used to obtain fine structure mapping information.

### On-surface nick-labelling and mapping of λ-DNA

**Figure 5:**
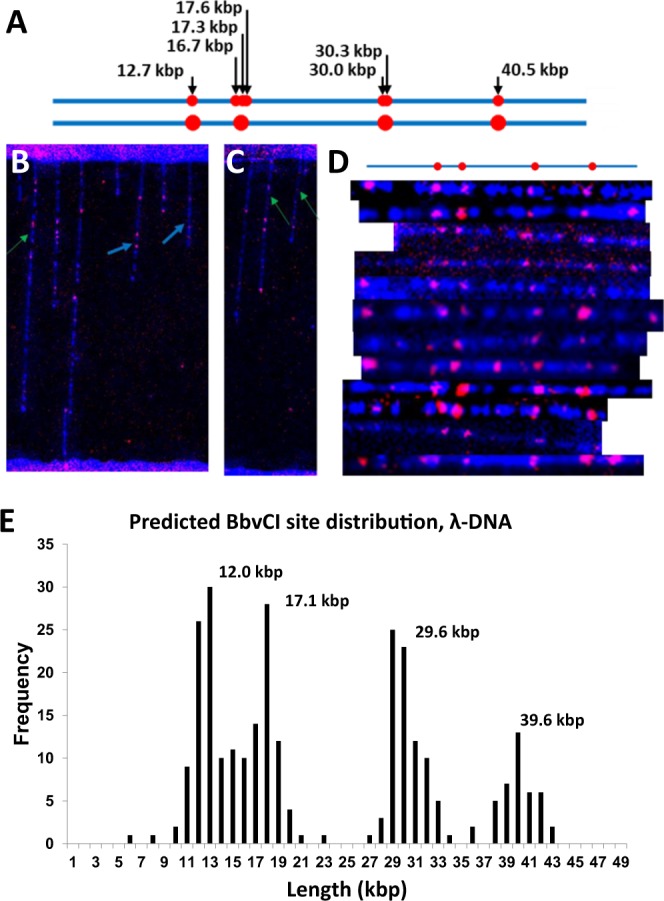
On-surface optical mapping of λ-DNA. (**A**) top: BbvCI site (red dots) distribution on λ-DNA (blue); bottom: simulated appearance of the BbvCI-map of λ-DNA (**B**,**C**) Microscope images of on-surface nick-labelled λ-DNA molecules that were concatemerized, combed, labelled, and stained. The green arrows point to λ-DNA molecules that contain the 4 BbvCI nick-labels and the blue arrows point to partially and/or weakly labelled molecules. (**D**) A collection of nick-labelled λ-DNA molecules aligned against the reference BbvCI map for λ-DNA (**E**) Histogram showing the predicted BbvCI nick-label positions on λ-DNA backbone. The predicted positions were found to be 12.0, 17.1, 29.6, and 39.6 kbp corresponding to the simulated label positions, 12.7, 17.1, 30.2, and 40.5 kbp respectively.

To demonstrate on-surface DNA mapping via fluorescent nucleotide incorporation, λ-DNA was used as a model genome and nick-labelled at the seven BbvCI sites (Fig. 5A (top), backbone is blue, BbvCI sites are marked red). Nicking was performed using Nb.BbvCI for 2 h at 37 °C followed by labelling with Klenow Fragment (3′ → 5′ exo-) at 37 °C for 2 h. After each reaction, the microliter-well was washed thoroughly with 1x CutSmart Buffer and 1x NEBuffer 2.0, respectively. Imaging was performed on a fully automated epi-fluorescence microscope, before and after staining with YOYO-1. Each addressed location on the micropatterned substrate was autofocused and imaged for ATTO-532 and for YOYO-1 successively, and the two images superimposed to produce a false-colour composite image (Fig. 5B–D). The octenyl sections appeared very bright due to the strong adsorption of YOYO-1. Figure 5B,C are raw images. The single λ-DNA molecules are combed starting from a random location in the 10 μm octenyl section. As can be observed in Fig. 2A,B, a substantial number of them combed beginning from the top of the octenyl section limiting the length of backbone available in PEG section for labelling. We therefore increased the chance of observing fully labelled λ-DNA spanning a PEG section by concatemerizing λ-DNA by briefly heating to 65 °C for 10 min followed by 1 h incubation at 37 °C. The green arrows in Fig. 5B,C point to individual λ-DNA molecules with the full BbvcI pattern, while the blue arrows indicate molecules with partial pattern. Nearly all the red labels observed colocalized with the DNA backbone confirming that the aggregated fluorophores are indeed incorporated ATTO-532 nucleotides. Staining with YOYO-1, we observed a discontinuous appearance of the DNA backbone (Fig. 5B–D). Although this did not affect our analysis for on-surface labelling experiments, it was interesting to note that the hydrogel properties influenced this effect. Higher percentage (7–10%) gels resulted in continuous appearance of backbone staining, while lower ones (3.3–4%) resulted in discontinuous appearance. However, we did not observe significant DNA labelling with higher percentage gels likely due to poor enzymatic access. It is also likely that relaxation of the linearized backbone in the presence of PEG moieties has caused these discontinuities^39^.

To begin analysis, we first identified labelled λ-DNA molecules by delineating a rectangle 60 px in height (corresponding to end-to-end distance between the most distant BbvCI sites, 27.8 kbp) and arbitrary width, to act as a reading frame, and randomly selecting molecules with at least 4 labels within the boundaries of the rectangle. In addition to the 4 nick-sites, linear λ-DNA possesses 12-bp 5′ overhangs on both ends that are also potential target sites for dye-dNTP incorporation. Some of the selected molecules are shown in Fig. 5D and were used to generate the histogram in Fig. 5E. The overall lengths of these DNA molecules were within an S.D. (3 μm) of each other. It can be observed that there are a few false positives, and most of the molecules did not have both the end-labels.

Inter-label distances for each molecule were measured and the values were normalized over the measured distance between the first (12.7 kbp) and fourth (40.5 kbp) nick-labels, thereby accounting for the overall variation in stretching factor (as evaluated and shown in Fig. 2C). These normalized inter-label measurements were plotted as a histogram (Fig. 5E) to illustrate the local variation in DNA stretching. A total of 81 molecules were selected with the above criteria. Molecules with end-label at 48.5 kbp in addition to the four BbvCI labels totalled 39 and were used to calculate the predicted positions of all BbvCI sites by adding the measured inter-site distances. Overall, the peaks (predicted site positions) match closely to the BbvCI site locations on λ-DNA.

This is the first report of on-surface fluorescent labelling and mapping of long DNA molecules that has the potential for adaption to high-throughput whole genome mapping, with the flexibility to perform multiple sequential enzymatic reactions on fixed DNA. Due to the high DNA density and average DNA lengths on our micropatterned substrate, we expect a significant increase in the amount of information that can be extracted than is currently possible. A calculation of DNA density using Fig. 5B yielded a loading density of 6.64 Gbp per sq. mm. Going further, we believe whole genome as well as targeted single DNA molecule interrogations are possible on this platform, such as multi-colour mapping and base-by-base sequencing. As noted earlier, stretching of λ-DNA was less uniform compared to extension in nanochannel arrays. However, the flexibility to perform multiple labelling steps on fixed DNA is highly significant and can open up new ways to analyse DNA sequence.

## Conclusions

Optical mapping is an advanced technique utilized at various levels of genomics, from genome assembly to disease marker discovery and diagnostics. Optical investigation of linear, surface-adsorbed DNA molecules is one of the two high-throughput means to mapping DNA. Here we report a novel substrate designed for improved DNA combing and single molecule analysis with the flexibility to perform sequence-specific cyclical enzymatic labelling chemistries. By chemically modifying a glass surface with two contrasting functionalities, octenyl and PEG, DNA ends are selectively bound to the hydrophobic octenyl section leaving the majority of DNA backbone spanning PEG sections leaving it accessible to enzymatic chemistry. The PEG section is designed to avoid overstretching of DNA molecules and to minimize free dye-nucleotide adsorption. We characterized DNA linearization on the micropatterned substrate and demonstrated enzymatic fluorescent labelling of single DNA molecules at sequence-specific sites. To this end, we showed that we could achieve on-surface transcription of T7 DNA molecules, as well as nick-labelling of λ-DNA at BbvCI sites. We believe our work could be extended to on-surface multi-colour cyclic chemistries, thereby opening new ways to investigate sequence information from Mbp-long DNA molecules in a high-throughput format.

## Methods

### Glass surface functionalization

Glass coverslips (22 × 22 mm) were used as substrates to covalently attach octenyl, PEG, and amine moieties via silanization reaction with 7-octenyltrimethoxysilane (OTMS), 2- [methoxy(polyethyleneoxy)6–9propyl]trimethoxysilane (PTMS), and 11-aminoundecyltriethoxysilane (AUTS) (Gelest), respectively. Glass cleaning and silanization procedures were modified from Labit *et al*.^40^, Naik *et al*.^41^ (OTMS) and Ho and Li^42^. Briefly, cleaned glass substrates were activated by treatment with either highly corrosive “piranha” solution or air plasma etching (Femto science, CUTE, 200 W 1–3 min). Activation exposed silanol groups on the glass surface, and under low-humidity conditions (<10% RH) reacted with the silane solution producing clear coatings of the respective functional groups. Reaction temperatures were between 21 and 23 °C^41^.

### Micropatterning surface functionalization

Micropatterning was performed in a class 10,000 cleanroom using positive photolithography. The fabrication process flow is shown in Fig. 1A, where the white-coloured substrate is glass, the olive-green layer represents octenyl, the red layer represents positive photoresist (PR), and the grey layer represents PEG. The octenyl-functionalized surface was coated with the positive PR (Microposit SC1813 or SC1827; Dow Corning), aligned underneath a photomask with the desired pattern, and exposed to UV light. The substrates were then developed using Microposit 351, dried using nitrogen, and loaded into the air-plasma etcher. Octenyl coating in the exposed regions on the substrate was etched away and the underlying glass was re-activated with silanol groups. Micropatterned substrates were loaded onto polypropylene coverslip racks, and the PR was stripped off the surfaces by sequential washing in acetone-isopropanol-water held inside an ultrasonic bath. After this, substrates were dried with filtered nitrogen gas and reacted with freshly-prepared PTMS solution in toluene and sealed under desiccating atmosphere.

Photomasks were designed using a CAD program and ordered from CAD/Art Services, Inc (Bandon, OR). A single pattern contained repetitive regions of inked and transparent bands with definite line widths and spacing. For example, one pattern consisted of 10 μm-wide inked lines with 40 μm-spacing, that we term ‘*10–40*’. Similarly, *10–10, 10–15, 20–90* and *40–170* patterns were also designed. The objective was to maximize the area of PEG sections containing combed DNA for fluorescence visualization, without loss in DNA combing density.

### High molecular weight DNA extraction

Mammalian cells were embedded in gel plugs and High Molecular Weight DNA was purified as described using a commercial large DNA purification kit (BioRad #170–3592). Plugs were incubated with lysis buffer and proteinase K for four hours at 50 °C. The plugs were washed and then solubilized with GELase (Epicentre). The purified DNA was subjected to 2.5 hours of drop-dialysis. It was quantified using the Quant-iT dsDNA Assay Kit (Life Technology), and the quality was assessed using pulsed-field gel electrophoresis^43^.

### DNA linearization by molecular combing

We performed molecular combing using a procedure similar to that first described in 1994 by Bensimon^15^. Briefly, we prepared DNA samples for molecular combing in 50 mM MES, 100 mM NaCl, pH 5.5–6.0 at concentrations ranging from 0.1 to 0.6 ng/µL. The substrate was first immersed into the DNA solution for a two-to-twenty-minute dwell time to allow the partially denatured tail ends to interact with the substrate. It was then withdrawn at a rate of 100 µm/s using a translational stage (Thorlabs MTS25-Z8).

### Flow-forced DNA linearization

Microchannels were fabricated in a process similar to that described by Karatekin *et al*.^44^. An SU-8 mould with channel widths ranging from 1 to 18 mm and heights ranging from 10 to 180 µm was fabricated. After casting PDMS, individual channels were cut out and fluid ports were bored with a biopsy punch. The face of the imprinted PDMS block was then air plasma treated and adhered to the functionalized substrate to create a liquid-tight flow cell. DNA was adsorbed and linearized using flow cells through a procedure similar to that described by Dimalanta *et al*.^33^. Briefly, 2–4 µL of YOYO-1-stained (100 nM) λ bacteriophage DNA (λ-DNA) in TE buffer (pH 8.0) was added into the flow cell port. The shear force exerted by the flowing buffer solution linearized the DNA as it adsorbed onto the positively charged amine surface.

### Hydrogel layer preparation and assembly

Polyacrylamide (PA) gel was used to affix combed DNA and maintain a stable aqueous environment around the DNA backbone. After combing DNA onto the micropatterned substrate, a low-adhesion PVC tape (Semiconductor Equipment Corp.) that was cut to the desired ‘microliter-well’ dimension was transferred onto the micropatterned substrate. This tape acted as a stencil delimiting the casting area of the gel. PA gel was prepared (4–10%) and pipetted at one-end of the microliter-well. A glass slide that was coated with the PVC tape was used to spread the gel droplet throughout the stencilled microliter-well area. After 5 mins of casting time, the slide and micropatterned substrate are gently separated from each other. The PA layer was then hydrated immediately with CutSmart 1x buffer, before preparing for the next step in device assembly.

### Microliter-well assembly for on-surface reactions

Enzymatic reactions were performed in two formats: (1) PDMS reaction wells assembled atop micropatterned substrate, and (2) PDMS-PMMA composite assembly on top of the substrate with a cast PA gel. PDMS slabs, that were cast in plastic dishes, were cut into approximately 12 × 20 mm blocks. PDMS was adhered to the functionalized substrate by either double-sided tape or plasma activation. PDMS adhered using double sided tape was first mated to a strip of double-sided tape and then an array of reaction wells was created using a 4 mm biopsy punch. PDMS adhered with plasma activation first had an array of wells punched out, followed by a 2-minute plasma treatment (Harrick Plasma, PDC-32G). DNA was combed onto functionalized substrates, allowed to dry at room temperature for 5 minutes, and the prepared PDMS well blocks were carefully positioned onto the targeted combing region. Each well was used for a unique experimental reaction condition. This microwell-format was used for reaction without a protecting hydrogel layer.

A PMMA sheet was laser cut to form the top and bottom layers of the device assembly, as well as to generate moulds for PDMS gaskets that will surround the gel region on the micropatterned substrate. The gaskets were mated first to the PMMA top layer and then placed over the gel-coated substrate. This assembly was then clamped to the PMMA bottom layer. The mouths of the microliter-wells are sealed with a tape, creating a tightly-sealed compartment for carrying out reactions.

### On-surface transcription on T7 DNA

T7 phage DNA (500 ng) was added into a combing reservoir containing 50 mM MES, 100 mM NaCl, pH 6.0 buffer and homogenized for 1 hour before combing onto *10–10* and *10–15* micropatterned substrates. Reaction wells were assembled as described above. Combed DNA molecules were rehydrated with rehydration buffer (0.1% BSA, 20 µM NTPs, 1 mM DTT, 5 mM MgCl2, 50 mM Tris, pH 7.8) for 2 minutes^18^. T7 RNA polymerase (RNAP) reaction buffer from New England Biolabs diluted to 1x concentration (40 mM Tris-HCl, 5 mM MgCl2, 1 mM DTT, pH 7.8) was then added to prime the same well for an additional minute. The master mix for the transcription reaction was prepared in a 0.6 ml microcentrifuge tube prior to pipetting into the well. The reaction mix contained 2.5 U of T7 RNAP, 10 µM Cy3-UTP, 200 µM NTPs, 100 µM DTT, 1 U/µL RiboGuard RNase inhibitor (Lucigen), 1x T7 RNAP reaction buffer. The mixture was gently pipetted into the well and the device was incubated in a humidified oven at 37 °C for 1 h. The well was evacuated and washed with 1x RNAP reaction buffer. The DNA backbone was stained with YOYO-1.

### On-surface nick-labelling of combed hgDNA

To ensure observation of full-length λ-DNA molecules within the PEG section (micropatterned regions grafted with PEG), DNA was concatemerized by heat-treating in 10 mM Tris-HCl buffer, pH 7.8, for 10 min at 65 °C followed by 1 h incubation at 37 °C. After this, DNA was suspended in a reservoir for combing onto a *10–40* substrate. PA gel was cast onto two demarcated microliter-well regions and a device was assembled as described in previous section. A nicking mix with 20 U of Nb.BbvCI (New England Biolabs) in 1x CutSmart buffer (New England Biolabs) was added onto the gel surface of one of the microliter-wells. In the control well, 1x Cutsmart buffer was added. The device was incubated at 37 °C for 2 h, after which both wells were evacuated and washed with 1x CutSmart buffer. Next, a labelling mix with 10 U of Klenow Fragment (3′ → 5′ exo-) (New England Biolabs), ATTO-532-dUTP (266 nM), and dATP/dGTP/dCTP (each 133 nM) in NEBuffer 2 (New England Biolabs) was added to both the wells. The labelling reaction was performed at 37 °C for 2 h, following which the wells were evacuated and washed with 1x NEBuffer 2 thoroughly before imaging. After acquiring a few images, 100 nM YOYO-1 solution was added to the wells to stain the DNA backbone for re-imaging.

### Image acquisition and analysis

We performed our imaging on a custom-built, semi-automated inverted fluorescent microscopy system. It includes a Rapid Automated Modular Microscope and Modular Infinity Microscope system (ASI) with an XYZ motorized stage (ASI, MS-2000), CRISP autofocus system (ASI), and high-speed filter wheel (Finger Lakes Instrumentation, HS-625) combined with a 100x oil-immersion objective (Olympus, UPlanSApo, NA = 1.40). We use diode-pumped solid-state laser light sources with 473 nm and 532 nm wavelengths (LASEVER, LSR473ML-100, LSR532ML-200), controlled through µManager (Open Imaging) using a custom-made TTL control system. Images were acquired with either iXon EMCCD (Andor, DU-888E-C00-#BV) or ORCA-Flash4.0 V2 CMOS (Hamamatsu, C11440). Data collected from our imaging system was processed on a computing cluster in ImageJ^45,46^ using previously developed computational methods and algorithms together with manual curation. Images were first processed to remove background and normalize signal intensity. Once processed, images were analysed semi-automatically using the Ridge Detection ImageJ plug-in^47^.
